# Diffuse Lung Metastases in *EGFR*-Mutant Non-Small Cell Lung Cancer

**DOI:** 10.3390/cancers11091360

**Published:** 2019-09-13

**Authors:** Subba R. Digumarthy, Dexter P. Mendoza, Atul Padole, Tianqi Chen, P. Gabriel Peterson, Zofia Piotrowska, Lecia V. Sequist

**Affiliations:** 1 Department of Radiology, Massachusetts General Hospital, Boston, MA 02114, USA; dpmendoza@mgh.harvard.edu (D.P.M.); apadole@mgh.harvard.edu (A.P.); 2 Department of Biostatistics and Computational Biology, Dana-Farber Cancer Institute, Harvard Medical School, Boston, MA 02215, USA; tianqi.chen.7@gmail.com; 3 Department of Radiology, Walter Reed National Military Medical Center and Department of Radiology and Radiological Sciences, Uniformed Services University of the Health Sciences, Bethesda, MD 20814, USA; pgpeterson@gmail.com; 4 Massachusetts General Hospital Cancer Center and Department of Medicine, Massachusetts General Hospital, Boston, MA 02114, USA; Zofia.Piotrowska@mgh.harvard.edu (Z.P.); LVSequist@partners.org (L.V.S.)

**Keywords:** epidermal growth factor receptor (*EGFR*) mutation, lung cancer, radiology

## Abstract

Diffuse lung metastases have been reported in non-small cell lung cancer (NSCLC) harboring epidermal growth factor receptor (*EGFR*) mutations. The purpose of our study was to compare the incidence of diffuse lung metastases in *EGFR*-mutant NSCLC and *EGFR*-wild type NSCLC and to assess other imaging features that may be associated with diffuse lung metastases in *EGFR*-mutant NSCLC. Two radiologists retrospectively reviewed pre-treatment imaging of metastatic NSCLC cases with known *EGFR* mutation status. We assessed the imaging features of the primary tumor and patterns of metastases. The cohort consisted of 217 patients (117 *EGFR*-mutant, 100 *EGFR* wild-type). Diffuse lung metastasis was significantly more common in *EGFR*-mutant NSCLC compared with wild-type (18% vs. 3%, *p* < 0.01). Among the *EGFR*-mutant group, diffuse lung metastases were inversely correlated with the presence of a nodule greater than 6 mm other than the primary lung lesion (OR: 0.13, 95% CI: 0.04–0.41, *p* < 0.01). *EGFR* mutations in NSCLC are associated with increased frequency of diffuse lung metastases. The presence of diffuse lung metastases in *EGFR*-mutant NSCLC is also associated with a decreased presence of other larger discrete lung metastases. *EGFR* mutations in NSCLC should be suspected in the setting of a dominant primary lung mass associated with diffuse lung metastases.

## 1. Introduction

Current guidelines recommend routine molecular testing for patients with metastatic non-small cell lung cancer (NSCLC) [1,2]. In those identified to have tumors with actionable somatic alterations, target-specific tyrosine kinase inhibitors (TKIs) are the preferred first-line treatment [1]. The most common such genetic alteration is a mutation in the epidermal growth factor receptor (*EGFR*) gene [3,4]. Five drugs targeting *EGFR*-mutant NSCLC are currently FDA-approved as front-line therapy [5,6,7,8,9]. 

This trend towards personalized, precision medicine in the treatment of NSCLC and the indispensable nature of imaging in the management of NSCLC have led to increased interest in radiogenomics and the correlation of radiologic features with genetic mutations. Several groups have investigated and reported the imaging findings of different genetic mutational subtypes in NSCLC [10,11,12,13,14,15]. Imaging features that have been associated with the primary tumor in *EGFR*-mutant NSCLC include peripheral location, pleural tagging, air bronchograms, and ground-glass opacities [16,17,18]. Several authors have also reported the increased frequency of diffuse lung metastases, also referred to as “miliary metastases” by some authors, in the setting of *EGFR* mutation-positive NSCLC [19,20,21]. 

To our knowledge, no study has investigated imaging features and patterns of metastases that may be associated with *EGFR*-mutant NSCLC with diffuse lung metastases. The goals of our study were to assess the incidence of diffuse lung metastases in *EGFR*-mutant and *EGFR* wild-type NSCLC on imaging and to assess other imaging features that may be associated with diffuse lung metastases in *EGFR*-mutant NSCLC. 

## 2. Results

### 2.1. Patients

The 217 patients studied included 117 patients with *EGFR*-mutant NSCLC and 100 patients with *EGFR* wild-type NSCLC and no documented driver mutation (Table 1). The median age was 65 years (range 26–90) and the majority was female (57%) and were current or previous smokers (59%). As expected, those with *EGFR*-mutant NSCLC were more likely to be female, never-smokers. 

The frequency of diffuse lung metastases was significantly higher in *EGFR*-mutant patients (18% vs. 3%, *p* < 0.01). *EGFR*-positive patients were also more likely to have nodal disease (*p* < 0.01), pleural metastases (*p* < 0.01), and adrenal metastases (*p* < 0.01).

### 2.2. Diffuse Lung Metastases in EGFR-Mutant NSCLC 

Among *EGFR*-mutant patients, we examined the characteristics of those with and without diffuse metastases (Table 2). The presence of a metastatic nodule >6 mm was inversely correlated with the presence of diffuse lung metastases (*p* < 0.01).

### 2.3. Multivariable Regression Model for Presence of Diffuse Lung Metastases in The Setting of EGFR-Mutation

Age (≥63, <63), zone, and lung metastases >6mm are significant predictors of whether patients had diffuse lung disease metastases or not. When holding the other covariates fixed, among *EGFR*-mutant patients, the odds of having diffuse lung metastases are 87% lower in those with lung metastases >6 mm than those without lung metastasis >6 mm (OR: 0.13, 95% CI: 0.04–0.41).

## 3. Discussion

Our findings add to the growing evidence that there is increased frequency of diffuse lung metastases in the setting of *EGFR*-mutant NSCLC. We also found that the presence of diffuse lung metastases in the setting of *EGFR*-mutant NSCLC is inversely correlated with the presence of larger discrete metastatic nodules.

In our cohort, there was almost a six-fold increased incidence (18% vs. 3%) of diffuse lung metastases in patients with metastatic *EGFR*-mutant NSCLC compared to patients with *EGFR*-wild type NSCLC. Other studies have reported rates of 12–50% [17,21,22]. Differences in frequencies among several studies may be, at least in part, due to the differences in defining the finding of diffuse lung metastases and miliary metastases. We adhered to a stricter definition (i.e., diffuse nodules ≤6 mm in size), while others have used the size cut-off of up to 30 mm [22].

Prior clinical- and population-based studies have also reported the increased frequency of “miliary metastases” in the setting of *EGFR*-mutant NSCLC [19,20,21]. Another retrospective study also reported higher incidence of *EGFR* mutations in those who present with miliary metastases compared to those who do not have miliary metastases [23]. While several authors have used the term “miliary” metastasis in the setting of *EGFR*-mutant NSCLC, we propose that the designation of “diffuse lung metastases” is more accurate. 

**Figure 1:**
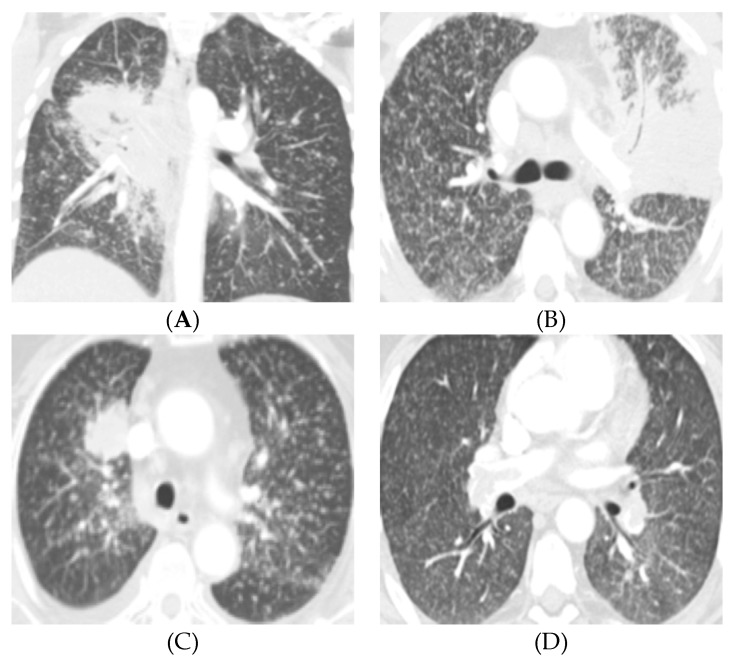
Four patients with *EGFR*-mutated lung cancers and diffuse lung metastases. (**A**) 44-year-old female never-smoker presents with a central right hilar mass with mediastinal and right hilar lymphadenopathy and innumerable 2–3 mm pulmonary nodules bilaterally. Patient was subsequently diagnosed with non-small cell lung cancer (NSCLC) with *EGFR* exon 19 mutation. Note the air bronchograms in the primary tumor, a feature also described in *EGFR*-mutated NSCLC. (**B**) 66-year-old male never-smoker presents with a consolidative mass in the left upper lobe and lingula with miliary-like pattern of diffuse 2–3 mm pulmonary nodules bilaterally and mediastinal and left hilar lymphadenopathy. This patient was found to have NSCLC with *EGFR* exon 21 mutation. Again, note the air bronchogram in the primary tumor. (**C**) 74-year-old female never-smoker presenting with a dominant right upper lobe mass and diffuse innumerable 2–3 mm nodules bilaterally. Patient had an *EGFR* exon 19 mutation. (**D**) 61-year-old male never-smoker presents with diffuse 2–3 mm lung nodules with a dominant 2.3 cm left lower lobe spiculated nodule (not shown), which was positive for *EGFR* exon 19 mutation.

Miliary nodular pattern of disease has distinct radiologic features characterized by diffuse, bilateral infiltration of the lungs by tiny, typically 1–4 mm in size, nodules likened to millet seeds [24]. This finding is seen in numerous infectious and inflammatory etiologies, including tuberculosis, histoplasmosis, silicosis, and sarcoidosis [25,26,27,28,29,30]. Miliary nodular patterns have also been in the setting of metastatic disease, most notably with primary thyroid cancer, renal cell carcinoma, and melanoma [24,25]. While the imaging features of diffuse lung metastases in *EGFR*-mutant NSCLC may overlap with the other pathologies that present with miliary nodules, *EGFR*-mutant NSCLC with diffuse lung metastases can by distinguished by the presence of a dominant primary lung mass or nodule (Figure 1). 

Of note, our findings also suggest that the presence of a larger (>6 mm) discrete lung metastasis is inversely correlated with diffuse lung metastases in the setting of *EGFR*-mutant NSCLC. It is unclear as to why those with diffuse lung metastases are less likely to have concomitant larger metastatic nodules, but it suggests a distinct mechanism of spread. We hypothesize that this may be due to the diffuse synchronous development of the nodules, which may lead to worse symptomatology and presentation before nodules have time to increase in size.

We did not find any other significant associations between the presence of diffuse lung metastases and tumor morphology, size, and location of primary tumor and the presence of lymphangitic carcinomatosis, pleural metastasis, and other distant metastases in the setting of *EGFR* mutations. Although some investigators have suggested that the exon-19 deletion subtype of *EGFR* may be associated with increased tendency for miliary metastases [23,31], our findings did not support this. A larger study cohort may be needed to validate these reports.

The single-institution, retrospective nature of our study predisposes it to selection bias and limits the findings’ generalizability to larger populations. Our relatively small cohort may also limit the study’s statistical power in discovering associations or lack thereof between diffuse lung metastases and other imaging features of *EGFR*-mutant NSCLC patients. Again, a larger cohort, perhaps from a collaborative multicenter study, would be helpful in validating our findings and in resolving these limitations. 

Although the presence of diffuse lung metastases in the setting of NSCLC may be suggestive of an underlying *EGFR* mutation, this feature does not replace molecular genotyping. Testing for *EGFR* mutations in NSCLC traditionally depended on unmodified Sanger sequencing, which requires at least 50% malignant cellularity to be reliable [2,32]. Subsequently, however, more sensitive PCR-based targeted methods have been developed and validated, requiring as little as 10% tumor content [2,33,34,35]. Although more sensitive, these methods still require adequate tissue for accurate diagnosis. Tissue sampling and subsequent testing can take time and potentially delay initiation of treatment. Another factor that can prolong time to diagnosis and treatment is the timing of testing. In many centers, including ours, it is still customary for the treating physician to decide if a tumor specimen should be tested for genetic mutations, as opposed to reflex testing, wherein a pathologist decides which specimens should be tested for genetic mutations, which can further delay diagnosis and treatment [36,37]. 

The presence of diffuse lung metastasis in the setting of NSCLC should increase the suspicion for the presence of *EGFR* mutation. This may be assistive in determining which patients may potentially benefit from expedited molecular testing and those who may benefit from repeat alternative testing following an unexpectedly negative or discordant result initial testing. 

## 4. Materials and Methods 

### 4.1. Patient Selection

Under an institutional “Partners Human Research” (the Massachusetts General Hospital IRB) review board-approved protocol (protocol number: 2019P000198), we searched our clinical database for patients with biopsy-proven stage IV NSCLC who had undergone genetic testing and found to have activating *EGFR* mutations between January 2010 and December 2013 and who also had CT and or PET/CT scans performed prior to any anti-cancer therapy either at our hospital or at an outside facility with the images uploaded in to our picture archiving and communication system. For controls, we selected a subset of NSCLC patients with genetic testing who were wild-type for *EGFR*, negative for other potentially targetable mutations (e.g., *ALK*, *ROS1*, *RET*, etc), and had similar pre-treatment imaging available for review. 

### 4.2. Genetic Analysis

Genotyping was performed using a multiplex PCR-based assay (SNaPshot^®^ platform, Applied Biosystems, Foster City, CA, USA). This system detects single nucleotide polymorphisms in 14 key cancer genes and more than 50 hotspot mutations [2,38]. The genetic testing was performed on formalin-fixed paraffin-embedded biopsy specimens that were obtained from different organs via surgical, bronchoscopic, or image guided percutaneous procedures. 

### 4.3. CT Imaging Protocol 

The CT examinations of the body (chest, abdomen, and pelvis) were performed on multidetector-row CT scanners with helical acquisition mode, automatic exposure control, tube potential 100–120 kV, slice thickness of 1–2.5 mm for chest and 5 mm for abdomen. The brain MR and/or CT images were reviewed for the presence of metastases. The 18-FDG PET images, when available, were reviewed to assess for metabolic activity and were correlated with CT images. 

### 4.4. Image Analysis

The cross-sectional imaging studies were independently reviewed by a board-certified thoracic radiologist (SRD) and a thoracic radiology fellow (DM, PGP), and discrepancies were resolved by consensus following concurrent review. The primary tumor was evaluated for size, lobar location, axial location within the lobe (inner, middle, or outer third), density (solid, pure ground glass opacity, or mixed density), cavitation, and air bronchograms. The metastatic lymph nodes were characterized as N0, N1, N2, or N3 per the American Joint Committee on Cancer (AJCC) 7th edition TNM staging manual [39]. More distant lymph nodes were classified along with distant metastases. The presence of metastases in the lung, pleura, adrenal glands, bones, soft tissues/viscera, and brain were documented for each patient. The presence of axial and septal interstitial thickening that involved greater than half a lobe and extending far beyond the primary tumor was considered as lymphangitic spread of tumor.

The lung metastases, when present, were classified as diffuse or discrete. Diffuse lung metastasis was defined as randomly distributed innumerable small nodules (less than or equal to 6 mm) of uniform size that were distributed over a wide area in both lungs (Figure 1). Diffuse lung metastases were evaluated for axial and craniocaudal distribution. Patients with metastatic lung nodules that did not meet the above criteria for diffuse pattern were further subdivided based on their location relative to the dominant lung mass as within the same lobe, ipsilateral different lobe, and contralateral lung. As most of the subjects presented prior to the adoption of the AJCC 8th edition in the United States and our institution, the criteria laid out in the AJCC 7th edition staging manual were used to designate TNM stage and overall stage category [39].

### 4.5. Statistical Analysis

Patient characteristics and imaging features were summarized using descriptive statistics. Continuous data were presented as medians with ranges, and categorical data were presented as frequencies with percentages. Comparisons were performed between *EGFR*-mutant and *EGFR* wild-type groups. Within the *EGFR*-mutant group, comparisons were also made between patients with diffuse lung metastases and those without diffuse lung metastases. Continuous characteristics between groups were compared using the Wilcoxon rank-sum test, and categorical features were compared using Fisher’s exact test. All tests were two-tailed, and *p*-values less than 0.05 were considered significant. 

In order to investigate the variables that can predict the presence of diffuse lung metastases among patients with *EGFR*-mutant patients, a multivariable logistic regression model was built. The criteria for choosing candidate predictors were *p*-value < 0.20 based on univariate analysis and proper sample size.

## 5. Conclusions

In conclusion, *EGFR*-mutant NSCLC is associated with increased frequency of diffuse lung metastases. The presence of this diffuse pattern in *EGFR*-mutant NSCLC is associated with decreased propensity for the presence of other non-miliary pulmonary nodules. *EGFR* mutations in NSCLC have important treatment and prognostic implications and should be considered in the setting of suspected NSCLC with diffuse lung metastases. Although these distinct features cannot replace molecular testing in determining the presence of *EGFR* mutations, they may help identify patients who may benefit from expedited or repeat testing following unexpectedly negative genotyping. 

## Figures and Tables

**Table 1:** Patients characteristics, tumor genotypes, and imaging features among all patients (*n* = 217).

Characteristics	All (*n* = 217)	*EGFR*		*p*-Value *
		Mutant (*n* = 117)	Wild-type (*n* = 100)	
Patients Characteristics				
			Median (range)	
Age	65 (26–90)	63 (26–90)	68 (42–84)	<0.01
Gender			*n* (%)	
Female	123 (57%)	81 (69%)	42 (42%)	<0.01
Male	94 (43%)	36 (31%)	58 (58%)	
Race				
Caucasian	185 (85%)	94 (80%)	91 (91%)	0.03
Asian	19 (9%)	12 (10%)	7 (7%)	
African-American	4 (2%)	3 (3%)	1 (1%)	
Hispanic	3 (1%)	3 (3%)	0 (0%)	
Others/Unknown	6 (3%)	5 (4%)	1 (1%)	
Smoking status				
Never	88 (41%)	72 (62%)	16 (16%)	<0.01
Ever	129 (59%)	45 (38%)	84 (84%)	
Primary tumor features				
Size (mm)	50 (10–134)	47 (11–134)	52 (10–115)	0.84
Primary lesion lobar zone				
Both	51 (24%)	29 (25%)	22 (22%)	0.18
Central	91 (42%)	54 (46%)	37 (37%)	
Peripheral	75 (35%)	34 (29%)	41 (41%)	
Solid				
No	21 (10%)	13 (11%)	8 (8%)	0.5
Yes	196 (90%)	104 (89%)	92 (92%)	
Air bronchograms				
No	156 (72%)	84 (72%)	72 (72%)	> 0.99
Yes	61 (28%)	33 (28%)	28 (28%)	
Cavity				
No	198 (91%)	111 (95%)	87 (87%)	0.05
Yes	19 (9%)	6 (5%)	13 (13%)	
Tumor Calcification				
No	211 (97%)	112 (96%)	99 (99%)	0.22
Yes	6 (3%)	5 (4%)	1 (1%)	
Nodal disease				
Negative	23 (11%)	19 (16%)	4 (4%)	<0.01
Positive	194 (89%)	98 (84%)	96 (96%)	
Metastatic sites				
Intrathoracic				
Absent	33 (15%)	21 (18%)	12 (12%)	0.26
Present	184 (85%)	96 (82%)	88 (88%)	
Pleura				
No	100 (46%)	70 (60%)	30 (30%)	<0.01
Yes	117 (54%)	47 (40%)	70 (70%)	
Lung				
No	67 (31%)	34 (29%)	33 (33%)	0.56
Yes	150 (69%)	83 (71%)	67 (67%)	
Diffuse lung				
No	193 (89%)	96 (82%)	97 (97%)	<0.01
Yes	24 (11%)	21 (18%)	3 (3%)	
Extrathoracic				
Absent	63 (29%)	33 (28%)	30 (30%)	0.88
Present	154 (71%)	84 (72%)	70 (70%)	
Bone				
No	133 (61%)	68 (58%)	65 (65%)	0.33
Yes	84 (39%)	49 (42%)	35 (35%)	
Brain				
No	140 (65%)	70 (60%)	70 (70%)	0.15
Yes	77 (35%)	47 (40%)	30 (30%)	
Adrenal				
No	170 (78%)	101 (86%)	69 (69%)	<0.01
Yes	47 (22%)	16 (14%)	31 (31%)	
Soft tissue				
No	179 (82%)	92 (79%)	87 (87%)	0.11
Yes	38 (18%)	25 (21%)	13 (13%)	

* *p*-Values provided are for comparison between *EGFR*-mutant and *EGFR*-wild type groups. Significant *p*-Values are highlighted.

**Table 2:** Patients characteristics and imaging features, among *EGFR*-mutant patients (*n* = 117).

Characteristics	All (*n* = 117)	Diffuse Lung Metastases		*p*-Value *
		Yes (*n* = 21)	No (*n* = 96)	
Patients characteristics				
			Median (range)	
Age	63 (26–90)	58 (37–82)	64 (26–90)	0.16
Size (mm)	47 (11–134)	47 (17–99)	47 (11–134)	0.42
Age		*n* (%)	*n* (%)	
<63	57 (49%)	14 (67%)	43 (45%)	0.09
≥63	60 (51%)	7 (33%)	53 (55%)	
Gender				
Female	81 (69%)	11 (52%)	70 (73%)	0.07
Male	36 (31%)	10 (48%)	26 (27%)	
Ethnicity				
Caucasian	94 (80%)	15 (71%)	79 (82%)	0.36
Asian	12 (10%)	3 (14%)	9 (9%)	
African American	3 (3%)	1 (5%)	2 (2%)	
Hispanic	3 (3%)	0 (0%)	3 (3%)	
Others/Unknown	5 (4%)	2 (10%)	3 (3%)	
Smoking status				
Never	72 (62%)	16 (76%)	56 (58%)	0.15
Ever	45 (38%)	5 (24%)	40 (42%)	
*EGFR* subtype				
Exon 19	61 (52%)	12 (57%)	49 (51%)	0.9
Exon 21	33 (28%)	5 (24%)	28 (29%)	
Exon 18	13 (11%)	1 (5%)	12 (13%)	
Exon 20	10 (9%)	3 (14%)	7 (7%)	
Primary tumor features				
Size >30 mm				
No	22 (19%)	3 (14%)	19 (20%)	0.76
Yes	95 (81%)	18 (86%)	77 (80%)	
Location				
Both	29 (25%)	9 (43%)	20 (21%)	0.11
Central	54 (46%)	8 (38%)	46 (48%)	
Peripheral	34 (29%)	4 (19%)	30 (31%)	
Solid				
No	13 (11%)	3 (14%)	10 (10%)	0.7
Yes	104 (89%)	18 (86%)	86 (90%)	
Air bronchograms				
No	84 (72%)	15 (71%)	69 (72%)	>0.99
Yes	33 (28%)	6 (29%)	27 (28%)	
Cavity				
No	111 (95%)	19 (90%)	92 (96%)	0.29
Yes	6 (5%)	2 (10%)	4 (4%)	
Calcification				
No	112 (96%)	21 (100%)	91 (95%)	0.58
Yes	5 (4%)	0 (0%)	5 (5%)	
Nodal disease				
Negative	19 (16%)	1 (5%)	18 (19%)	0.19
Positive	98 (84%)	20 (95%)	78 (81%)	
Metastases sites				
Pleural				
No	70 (60%)	11 (52%)	59 (61%)	0.47
Yes	47 (40%)	10 (48%)	37 (39%)	
Extra-thoracic				
Absent	33 (28%)	6 (29%)	27 (28%)	>0.99
Present	84 (72%)	15 (71%)	69 (72%)	
Bone				
No	68 (58%)	12 (57%)	56 (58%)	>0.99
Yes	49 (42%)	9 (43%)	40 (42%)	
Brain				
No	70 (60%)	11 (52%)	59 (61%)	0.47
Yes	47 (40%)	10 (48%)	37 (39%)	
Adrenal				
No	101 (86%)	17 (81%)	84 (88%)	0.48
Yes	16 (14%)	4 (19%)	12 (12%)	
Soft tissue				
No	92 (79%)	17 (81%)	75 (78%)	>0.99
Yes	25 (21%)	4 (19%)	21 (22%)	
Other lung metastasis >6 mm				
No	49 (42%)	16 (76%)	33 (34%)	<0.01
Yes	68 (58%)	5 (24%)	63 (66%)	

* *p*-Values provided are for comparison between *EGFR*-mutant NSCLC patients with diffuse lung metastases versus those without diffuse lung metastases. Significant *p*-Values are highlighted.
